# Optimizing the Postcataract Patient Journey Using AI-Driven Teleconsultation: Prospective Case Study

**DOI:** 10.2196/72574

**Published:** 2025-08-18

**Authors:** Joukje C Wanten, Noël J C Bauer, Mohita Chowdhury, Aisling Higham, Nick de Pennington, Frank J H M van den Biggelaar, Rudy M M A Nuijts

**Affiliations:** 1 University Eye Clinic Maastricht, Maastricht University Medical Centre+, P Debyelaan 25, Maastricht, 6229HX, The Netherlands, 31 0433871594; 2 Ufonia Limited, Oxford, United Kingdom

**Keywords:** algorithm-based screening, remote patient monitoring, postoperative follow-up, telemedicine, digital health, eHealth intervention, prospective clinical study

## Abstract

**Background:**

Given the increasing global demand for ophthalmologic care and the anticipated shortage of ophthalmology professionals, innovative solutions are essential for optimizing health care delivery. Digital health technologies offer promising opportunities to efficiently manage high patient volumes. Cataract surgery, with its established safety profile and routine postoperative care, provides an ideal setting for implementing such innovations. Structured clinical questions have proven effective in identifying patients requiring further assessment, supporting the feasibility of follow-up through telephone consultations. To further extend this approach, artificial intelligence–based follow-up systems may offer an opportunity to automate these interactions, reduce clinician workload, and streamline care pathways.

**Objective:**

The aim of the study is to assess the clinical safety and effectiveness of an artificial intelligence–based follow-up call system (Dora-NL1) in identifying patients who require further assessment after cataract surgery in the Netherlands.

**Methods:**

This prospective single-center study was conducted at the University Eye Clinic Maastricht, the Netherlands. Adult patients who underwent uncomplicated cataract surgery were eligible to participate. All patients received a Dora-NL1 follow-up telephone call at 1 and 4 weeks postoperatively in addition to standard care (a clinician-led telephone consultation at week 1 and an in-person hospital visit at week 4). The Dora-NL1 calls used a standard conversational flow to evaluate symptoms and recommend a clinical outcome. The recommended outcomes of Dora-NL1 were based on the symptoms reported by the patient. Clinical safety and accuracy were assessed by comparing Dora-NL1 outcomes to blinded clinician assessments of recorded calls and to standard postoperative care. Patient-reported usability was measured using the Telehealth Usability Questionnaire and Net Promoter Score.

**Results:**

A total of 105 patients with a mean age of 72 (SD 7) years were included in the analysis. Dora-NL1 demonstrated high agreement with clinician-supervised calls, with symptom evaluation accuracy ranging from 89% to 99% (κ=0.390‐0.947) and care management decision accuracy between 83% and 88% (κ=0.640‐0.753). At week 1, Dora-NL1 showed a sensitivity of 100% and a specificity of 42% compared to standard clinician-led telephone consultations with no missed clinical concerns. At week 4, compared to the in-person follow-up, Dora-NL1 failed to identify 4 (4.1%) patients who required unexpected management changes, including 3 with asymptomatic complications detected only via slit lamp examination and 1 with complaints in the nonoperated eye. Patients rated Dora-NL1 positively, with Net Promoter Scores of +13.5 and +12.6 at week 1 and 4, respectively. The Telehealth Usability Questionnaire was completed by 98 patients, yielding a mean score of 3.19 (SD 1.13) on a 5-point scale, highlighting its simplicity, ease of use, and audibility.

**Conclusions:**

Dora-NL1 is a safe and effective tool for automated postoperative screening following cataract surgery. It offers a safe alternative to clinician-led telephone consultations in routine cases but cannot fully replace in-person examinations.

## Introduction

Our health care systems worldwide face increasing challenges, driven in large part by demographic shifts such as population aging and the rising prevalence of chronic diseases. These developments contribute to a growing mismatch between demand for and supply of health care services. In ophthalmology, a field characterized by high patient volumes and an aging patient population, it is expected that, by 2035, the workforce will be insufficient to meet the growing demand for care [1-3]. This trend is expected to intensify the burden on our health care systems. Moreover, the strain extends beyond ophthalmology, with similar challenges observed in most specialties including cardiology, urology, and neurology, underscoring the need for innovative, scalable solutions to maintain the quality and accessibility of care [2]. To mitigate this growing pressure, efficient management of high-volume workloads is essential. eHealth solutions offer promising opportunities to optimize health care delivery, enhance accessibility, and reduce the burden on clinical personnel. The COVID-19 pandemic further accelerated the adoption of such technologies, demonstrating their potential to ensure continuity of care and support remote follow-up [4,5]. In ophthalmology, advances in digital or remote health technologies have facilitated developments in triage, diagnosis, and patient monitoring [4-6]. Cataract care, in particular, is well suited to such innovations, as it is among the most frequently performed procedures worldwide and associated with a high safety profile and predictable recovery [7,8]. Previous studies have shown that structured sets of postoperative clinical questions can reliably identify patients who require further assessment within the first week after surgery. This has enabled safe and feasible alternatives to in-person follow-up during the early postoperative period, such as nurse-led telephone consultations [9,10]. Building on these developments, automated systems offer an additional opportunity to streamline care pathways. One such innovation is the voice-based automated conversational assistant, “Dora” (Ufonia Limited), which uses a machine learning–driven conversational model to conduct follow-up after cataract surgery via telephone. The system applies a standardized script of clinically validated questions to screen for symptoms that may indicate postoperative complications. Only patients who give responses requiring further attention are flagged for clinical review, potentially reducing the need for in-person or clinician-led consultations. Dora has already been validated for use in the United Kingdom, where it has demonstrated safety, feasibility, and high patient acceptability while also reducing the burden on outpatient services [11]. The English Dora system uses a discriminative dialogue mechanism that combines rule-based natural language processing with machine learning components. It uses a Dual Intent and Entity Transformer classifier for intent recognition [12]. This model interprets patient responses by mapping them to predefined “intents,” which in turn determine the next action in the dialogue flow. These “intents” were carefully designed to capture relevant aspects of postoperative symptomatology and are linked to a rule-based system that ensures that the complete sequence of questions is asked in a clinically appropriate order. Both the conversational flows and response content were developed in close collaboration with specialist clinicians and have been previously described in detail [11].

In this study, our aim is to validate and evaluate the clinical safety and effectiveness of the first Dutch version (Dora-NL1) in identifying patients who require further assessment after cataract surgery in the Netherlands. We hypothesize that the Dora-NL1 will be a safe and effective tool for early complication screening and a reliable alternative to human-led telephone consultation in routine cataract care.

## Methods

### Recruitment

Adult patients (≥21 years) who underwent uncomplicated cataract surgery on either 1 or both eyes, with implantation of a monofocal (including toric) intraocular lens (IOL) targeted for emmetropia, were recruited for this study at the University Eye Clinic of the Maastricht University Medical Center+. Patients were excluded if they had serious ocular comorbidities requiring face-to-face postoperative visits, underwent combined procedures (eg, glaucoma or retinal surgeries), needed follow-up visits within 1 week due to complications, were not able to speak Dutch, or had significant hearing or cognitive impairments.

### Ethical Considerations

All patients were informed about the study and signed an informed consent before enrollment. This prospective validation study was approved by the local medical ethics committee (Medisch-ethische toetsingscommissie azM/UM, Maastricht, the Netherlands; approval METC 2022-3258) and executed in accordance with the Declaration of Helsinki. Patient data were anonymized to ensure confidentiality. Participants did not receive financial compensation for their involvement.

### Dutch Model Development and Validation

**Figure 1.**
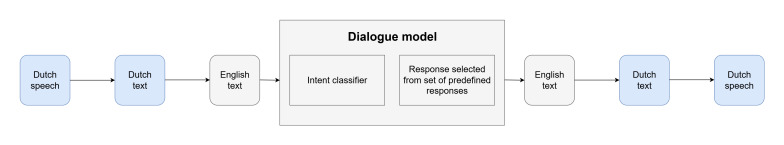
Schematic workflow of the Dora-NL1 system.

The model for the autonomous cataract follow-up call system, Dora, was originally developed for English-speaking patients. In this study, the model was adapted for use in Dutch, with the initial Dutch system developed from the pretrained English version. To facilitate this adaptation, an intermediary translation function was integrated into the Dora-NL1 software, translating captured Dutch answers into English, formulating a response, and then converting it back to Dutch for speech output, as presented in Figure 1. While this is a crucial step, the translation process introduces complexity and the risk of altering the original message. The Dora model has a finite number of potential utterances; thus, it was possible to confirm that these were correctly translated into Dutch. In addition to the translation layer, the system was further adapted to improve its handling of Dutch-language input. Dutch utterances that were not recognized by the English Dora system were translated into English and manually reviewed to assign them to the appropriate intent category. These additions were incorporated into the training data to enhance the model’s ability to interpret Dutch responses. All translations and validation of the Dutch utterances were carried out by a bilingual clinician-researcher who was independent of the Dora development team. To ensure the reliability of the Dora-NL1 software, we ran a preliminary investigation before starting the study by looking at the agreement between Dutch (Dora-NL1) and English (Dora-R2) calls. In total, 12 different postoperative scenarios were created, reflecting both the common and most severe outcomes of patients who underwent cataract surgery. These scenarios were simulated by the bilingual clinician-researcher in both languages using a predefined strict script that underwent forward and back-translation and independent expert review to ensure accuracy and consistency. The responses from both models were compared to assess their consistency, defined as acceptable when no statistically significant differences were found in symptom assessment and care management decisions. The questions and responses used for model validation were similar to those used in the trial version of the Dora-NL1. Furthermore, before starting the trial, we recruited volunteers to evaluate the different regional accents and variations in phrasing.

### Postoperative Follow-Up

**Figure 2.**
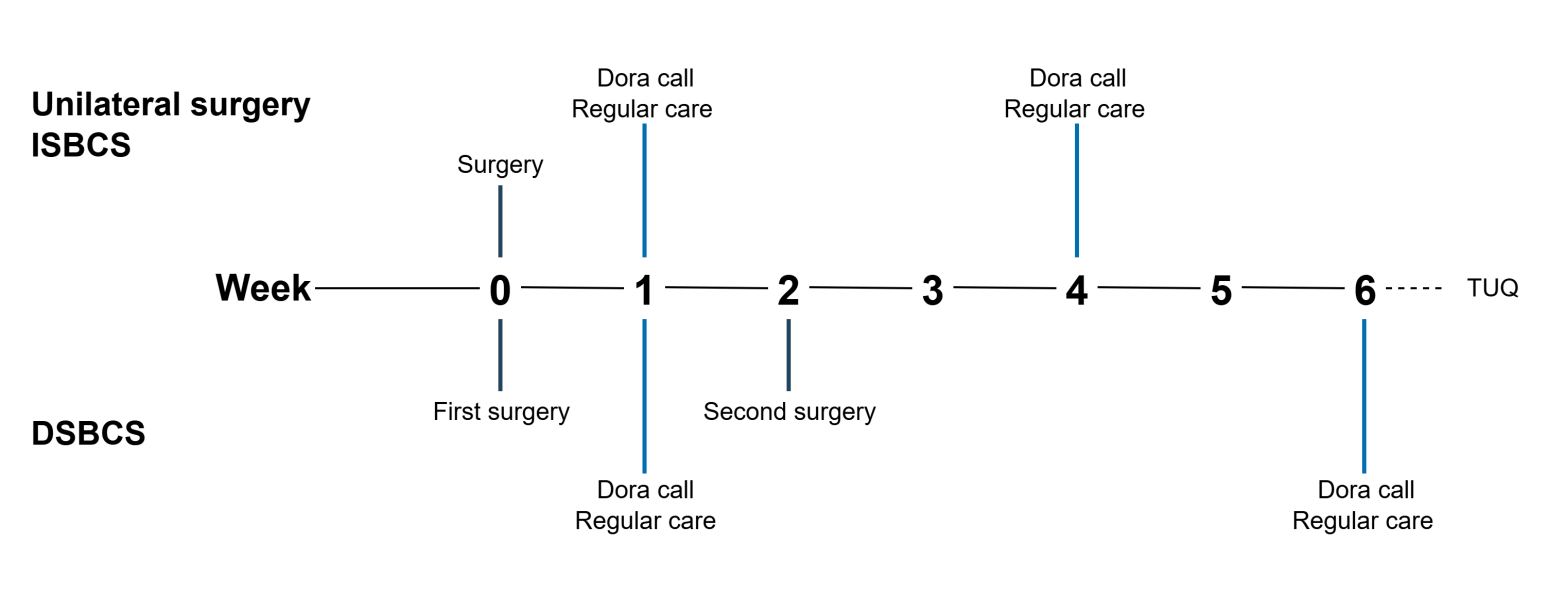
Patient pathway. DSBCS: delayed sequential bilateral cataract surgery; ISBCS: immediate sequential bilateral cataract surgery; TUQ: Telehealth Usability Questionnaire.

Patients undergoing bilateral cataract surgery underwent either immediate sequential bilateral or delayed sequential bilateral cataract surgery. For those undergoing unilateral surgery, the postoperative follow-up schedule aligned with that of immediate sequential bilateral cataract surgery. All patients received regular postoperative care in addition to the Dora-NL1 calls, as shown in Figure 2. One week after the first eye surgery, patients received both a Dora-NL1 call and a regular postoperative telephone consultation with a clinician. During the telephone consultations, a standardized set of questions was used to evaluate the patient’s symptoms and identify any necessary management changes. Management changes included deviations from the standard eye drop tapering schedule, the addition of any drops (excluding artificial tears), the need for an additional hospital visit, or the implementation of further interventions (excluding suture removal). Clinicians conducting the telephone consultations were masked to the results of the Dora-NL1 calls. For patients receiving a toric IOL, a 1-week follow-up hospital visit was scheduled instead of a telephone consultation. At 4 weeks postoperatively, another Dora-NL1 call was conducted within 24 hours before the regular outpatient clinic visit. The final postoperative visit at the outpatient clinic consisted of a routine ophthalmological assessment, including evaluation of the refractive status and visual acuity as well as slit lamp and retinal examination.

### Dora-NL1 Call

The postoperative Dora-NL1 call followed a structured conversational flow: greeting and introduction, patient identification, symptom evaluation, patient queries, care management decision-making, user-acceptability question, and call closure. The key symptoms that were assessed included redness, pain, reduced vision, flashing lights, and floaters. At the core of this, Dora decision-making is an intent recognition system, a Dual Intent and Entity Transformer–based classifier that classifies the patient responses into predefined categories of intents (such as a “red eye” or “poor vision”) [12-14]. These intent categories have been developed based on 2 prior clinical studies, and real-world use of Dora in the United Kingdom, with the input of clinicians and system developers [11,15]. For each patient utterance, the classifier assigns a confidence score reflecting the system’s certainty in its interpretation. If the confidence score for a response falls below the preset threshold, Dora-NL1 prompts the patient to repeat or rephrase their response—allowing up to 2 attempts—to improve the accuracy of symptom detection.

Dora-NL1 classified each reported symptom as either absent, present without clinical significance, present with clinical significance, or inconclusive due to insufficient information. The thresholds used to flag “potential concerns” were intentionally set to err on the side of caution. These were based on the framework established for the English version and were informed by clinical expertise and relevant literature on symptomatology, with a focus on sensitivity to minimize the risk of missing significant complications. If there was incomplete information regarding a symptom or if the patient asked a question that Dora-NL1 could not address, the overall call outcome defaulted to “potential clinical concerns.”

Based on the overall symptom evaluation, Dora-NL1 provided an overall outcome of either “no clinical concerns” or “potential clinical concerns.” The decision-making flowchart can be found in Multimedia Appendix 1. Depending on the overall call outcome, Dora-NL1 generated a corresponding care management recommendation: either “no review recommended” or “clinician review recommended.” All Dora-NL1 calls were recorded and subsequently reviewed by a clinician, who independently assessed symptoms and made clinical decisions based on the audio recordings while masked to Dora-NL1’s outputs and the regular care outcomes.

### User Acceptability

At the end of each call, Dora asked patients to rate their likelihood of recommending the automated system to others on a scale of 1 to 10. The Net Promoter Score (NPS) was calculated by subtracting the “%-detractors” (scores of 1 to 6) from the “%-promoters” (scores of 9 or 10) [16]. Additionally, after the final postoperative visit, patients were invited to complete the Telehealth Usability Questionnaire (TUQ), translated to Dutch. The TUQ consists of 20 questions that can be answered on a 5-point scale ranging from 1=fully disagree to 5=fully agree [17].

### Sample Size and Statistical Analysis

For this pilot study, a sample size of 100 participants who completed the 1-week call was used. Data analysis was conducted using SPSS (version 28.0; IBM Corp) and Microsoft Excel. Qualitative variables were summarized as frequencies and percentages, while descriptive statistics, including mean and SD, were calculated for quantitative variables. The agreement between the English and Dutch models in the developmental phase was tested using chi-square or Fisher exact tests. The interobserver reliability was calculated using the Cohen κ coefficient. The accuracy of the tool was defined in accuracy, specificity, and sensitivity, including 95% CIs of these proportions calculated using the Wilson score interval. A significance level of ≤.05 was applied.

## Results

### Preliminary Study Results

The agreement between the Dutch (Dora-NL1) and English (Dora-R2) versions used 12 scenarios. The accuracy in terms of detecting symptoms, including redness, pain, floaters, and flashing lights, was 100%. With respect to reduced vision, the accuracy was 83.3%, affected by 2 missing data points where Dora was unable to collect or understand the patient’s response (1 from the English model and 1 from the Dutch model). The accuracy of the care management decisions was 91.7%, with the decrease attributed to these missing data points. There was no statistically significant difference found between the 2 language models (*P*=.32). Detailed responses for each scenario are provided in Multimedia Appendix 2.

### Study Results

**Figure 3.**
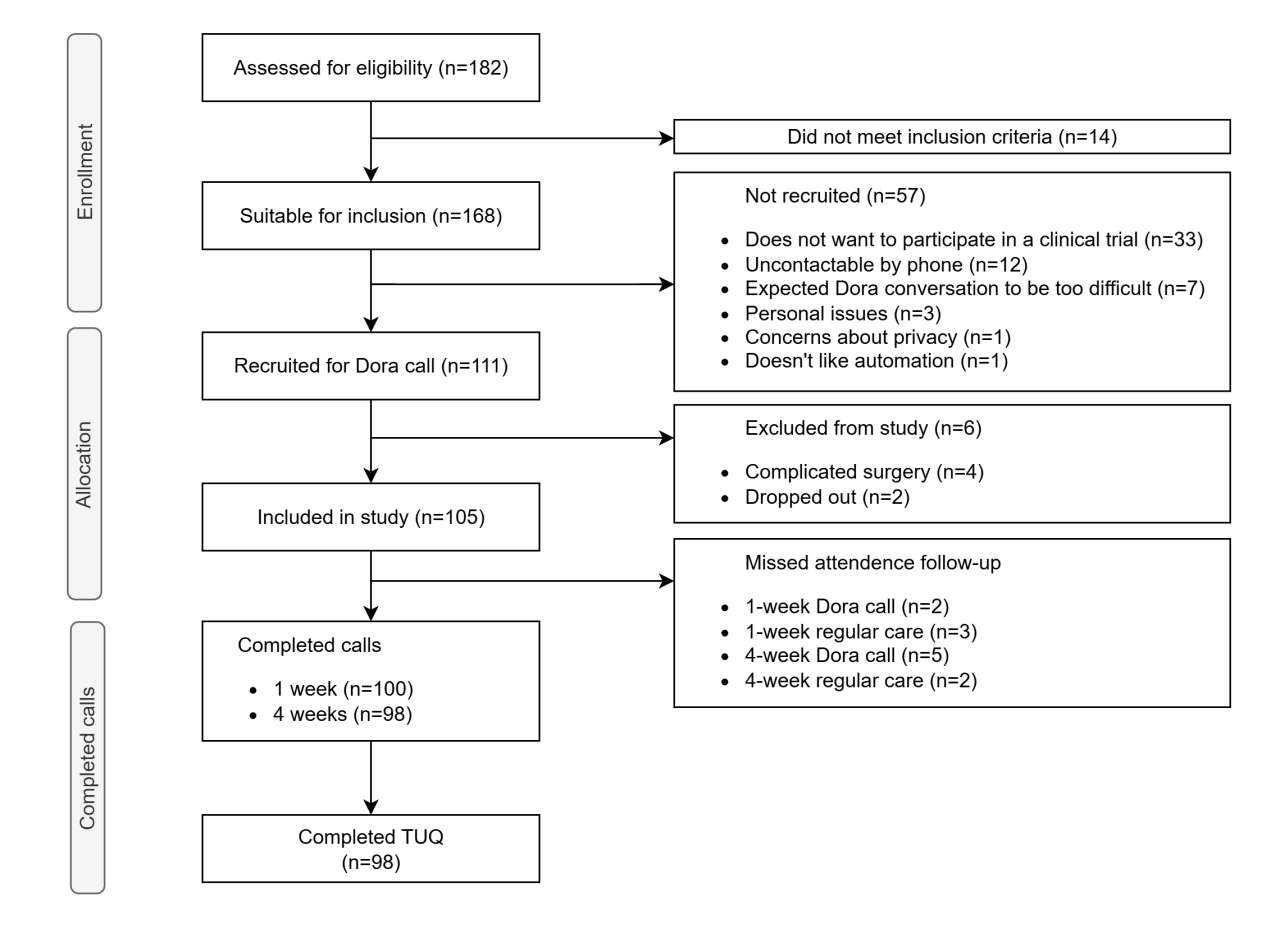
Flowchart study. TUQ: Telehealth Usability Questionnaire.

Between July 2023 and April 2024, a total of 182 patients who were scheduled for cataract surgery were screened for eligibility. Of these, 168 were suitable for participation, and 111 patients provided informed consent. In total, 105 patients (190 eyes) with a mean age of 72 (SD 7; range 50‐84) years were included in the analysis; 100 patients completed the 1-week follow-up with Dora-NL1, and 98 patients completed the 4-week follow-up. The patient flowchart can be found in Figure 3. The baseline characteristics are summarized in Table 1.

**Table 1.** Baseline characteristics.

Demographic variables	Participants (N=105), n (%)
Sex, n (%)	
Male	44 (41.9)
Female	61 (58.1)
Surgical risk factors, n (%)	
High myopia	3 (2.9)
High hyperopia	3 (2.9)
Glaucoma	2 (1.9)
Corneal pathology	7 (6.7)
Diabetic retinopathy	1 (1.0)
Previous vitrectomy	3 (2.9)
Type of surgery, n (%)	
Unilateral	20 (19.0)
DSBCS^a^	29 (27.6)
ISBCS^b^	56 (53.3)
IOL^c^ characteristics (n=190)	
Type of IOL, n (%)	
Monofocal nontoric	169 (88.9)
Monofocal toric	21 (11.1)
IOL power (diopter), mean (SD)	20.0 (3.8)
Refractive target (diopter), mean (SD)	−0.03 (0.12)

a DSBCS: delayed sequential bilateral cataract surgery.

b ISBCS: immediate sequential bilateral cataract surgery.

c IOL: intraocular lens.

### Postoperative Week 1

The agreement between the assessments made by Dora-NL1 at week 1 and those made by the clinician reviewing the Dora-NL1 call is shown in Table 2. The sensitivity ranged from 66.7% to 100%, and specificity ranged from 78.6% to 97.4% in identifying key symptoms and making care management decisions, with κ values ranging from 0.390 to 0.947. Only the detection of new floaters at week 1 showed a sensitivity of 66.67% and a specificity of 96.81%. Sensitivity was reduced because Dora failed to capture 2 patients’ initial reports of floaters. In line with this protocol to repeat the question when the input is unclear or not captured by the system, Dora reasked the patients whether they had floaters. On the second attempt, both responded that they did not, resulting in an incorrect final classification. The agreement between the automated care management decisions made by Dora-NL1 and the regular care is shown in Table 3. At week 1, only patients who received regular telephone consultations were analyzed, excluding those with toric IOLs (n=13) who were scheduled for routine postoperative hospital visits. At this time, Dora-NL1 demonstrated 100% sensitivity with no false negatives and a specificity of 41.7%, yielding a false positive rate of 57.5%.

**Table 2.** Symptom and outcome accuracy of automated Dora versus supervised Dora call at week 1.

Decision (n=100)	Accuracy^a^ (%) (95% CI)	Sensitivity (%) (95% CI)	Specificity (%) (95% CI)	κ (95% CI)	*P* value
Redness^b^	96.0 (90.12 to 98.4)	100 (56.6 to 100.0)	95.79 (89.7 to 98.4)	0.695 (0.416 to 0.973)	<.001
Pain^c^	97.0 (91.6 to 99.0)	100 (20.7 to 100.0)	96.97 (91.5 to 99.0)	0.390 (−0.149 to 0.929)	<.001
Vision issue^c^	94.0 (87.5 to 97.2)	100 (70.1 to 100.0)	93.41 (86.4 to 96.9)	0.718 (0.508 to 0.928)	<.001
New floaters	95.0 (88.8 to 97.9)	66.7 (30.0 to 90.3)	96.81 (91.0 to 98.9)	0.589 (0.262 to 0.916)	<.001
Flashing lights	98.0 (93.0 to 99.5)	100 (86.2 to 100.0)	97.37 (90.9 to 99.3)	0.947 (0.874 to 1.000)	<.001
Outcome	88.0 (80.2 to 93.0)	100 (92.0 to 100.0)	78.57 (66.2 to 87.3)	0.763 (0.641 to 0.885)	<.001

a Accuracy=(true positive+true negative)/(true positive+true negative+false positive+false negative).

b Amount of cases with “incomplete information”: n=4.

c Amount of cases with “incomplete information”: n=1.

**Table 3.** Accuracy in clinical decision-making between automated Dora versus regular telephone consultation at week 1^a^.

Dora decision	Decision during the regular telephone consultation		Total, n (%)
	Potential concerns, n (%)	No clinical concerns, n (%)	
Potential concerns	1 (1.1)	50 (57.5)	51 58.6)
No clinical concerns	0 (0)	36 (41.4)	36 (41.4)
Total	1 (1.1)	86 (98.8)	87 (100)

a Sensitivity: 100% (95% CI 20.7-100.0), specificity: 41.9% (95% CI 32.0-52.4), κ: 0.016 (95% CI 0.00-0.398); *P*=.39.

### Postoperative Week 4

Table 4 summarizes the agreement between the Dora-NL1 assessments at week 4 and those by the clinician reviewing the Dora-NL1 call, with a 100% sensitivity and specificity ranging from 88.3% to 99% for symptom evaluation. Despite this agreement, 3 asymptomatic patients who were identified as having “no clinical concerns” required an unexpected management change at the 4-week postoperative clinic visit, and 1 patient who had been asymptomatic of visual concerns at the week 1 Dora and clinician calls reported binocular diplopia. The 3 asymptomatic patients all had inflammatory cells detected during slit lamp examination, with 1 of these patients also having a residual lens fragment in the anterior chamber. They received topical corticosteroids and required additional follow-up visits. The fourth patient who reported binocular diplopia was evaluated by an orthoptist, who identified monocular diplopia in the contralateral eye due to postoperative changes after macular pucker peeling.

**Table 4.** Symptom and outcome accuracy of automated Dora versus supervised Dora call at week 4.

Decision (n=98)	Accuracy^a^ (%) (95% CI)	Sensitivity (%) (95% CI)	Specificity (%) (95% CI)	κ (95% CI)	*P* value
Redness^b^	99.0 (94.4-99.8)	100 (34.2-100.0)	99.0 (94.3-99.8)	0.795 (0.403-1.000)	<.001
Pain^c^	98.0 (92.9-99.4)	100 (34.2-100.0)	97.9 (92.7-99.4)	0.657 (0.216-1.000)	<.001
Vision issue	96.9 (91.4-99.0)	100 (56.6-100.0)	96.8 (90.9-98.9)	0.754 (0.487-1.000)	<.001
New floaters^d^	88.8 (81.0-93.6)	100 (51.0-100.0)	88.3 (80.3-93.3)	0.823 (0.656-0.990)	<.001
Flashing lights	94.9 (88.6-97.8)	100 (77.2-100.0)	94.1 (87.0-97.5)	0.809 (0.648-0.970)	<.001
Outcome	82.7 (74.0-88.9)	93.6 (79.3-100.0)	77.6 (66.3-85.9)	0.640 (0.491-0.789)	<.001

a Accuracy = (true positive+true negative ) /(true positive+true negative+false positive+false negative).

b Amount of cases with “incomplete information”: n=1.

c Amount of cases with “incomplete information”: n=3.

d Amount of cases with “incomplete information”: n=2.

### Acceptability and Patient Experiences

Patients rated their likelihood of recommending the Dora-NL1 calls, with mean scores of 7.59 (SD 1.77) at week 1 and 7.38 (SD 2.35) at week 4, shown in Table 5. The corresponding NPSs for these calls were +13.5 and +12.6, respectively. The TUQ (n=98) had an overall mean score of 3.19 (SD 1.13). High scores were given for aspects such as time savings due to reduced travel (mean 3.80, SD 1.11), simplicity (mean 3.89, SD 0.96), ease of use (mean 3.94, SD 0.88), and audibility (mean 3.97, SD 0.91). However, the user experience of Dora-NL1 was not deemed equivalent to an in-person consultation with a clinician, with a mean score of 2.46 (SD 1.07). Detailed questionnaire results are available in Multimedia Appendix 3.

**Table 5.** Net Promoter Scores of Dora-NL calls at 1 and 4 weeks postoperatively.

Net Promoter Score	Week 1 (n=96), n (%)	Week 4 (n=95), n (%)
1	1 (1)	3 (3.2)
2	1 (1)	3 (3.2)
3	1 (1)	3 (3.2)
4	4 (4.2)	3 (3.2)
5	5 (5.2)	6 (6.3)
6	6 (6.3)	7 (7.4)
7	17 (17.7)	13 (13.7)
8	30 (31.3)	20 (21.1)
9	23 (24)	22 (23.2)
10	8 (8.3)	15 (15.8)

## Discussion

### Principal Findings

The purpose of this study was to evaluate the clinical safety and effectiveness of the Dutch Dora-NL1 model in identifying patients who require further assessment following uncomplicated cataract surgery. Our findings demonstrated high overall accuracy of Dora-NL1’s symptom evaluation and care management decision-making at both 1 and 4 weeks after surgery when compared to the clinician-supervised Dora-NL1 calls. At week 1, Dora-NL1 exhibited a perfect sensitivity of 100% for care management decision-making when compared to a regular telephone consultation. However, at week 4, clinical issues in 4 asymptomatic patients were identified during face-to-face consultations, which were not detected by Dora or by the clinician reviewing the calls. In terms of user acceptability, most patients expressed a positive attitude toward the tool, highlighting its simplicity, ease of use, and audibility.

The Dora-NL1 model was designed to err on the side of caution, thereby ensuring that patients classified as having “no concerns” by Dora are unlikely to have underlying clinical issues. Additionally, in cases of “incomplete information,” patients were assumed to have potential concerns, which may have increased the rate of false positives and, with that, lowered the specificity. The sensitivity and specificity of the automated Dora-NL1 outcomes compared to the supervised Dora-NL1 calls were high both at 1 and 4 weeks. Only the sensitivity for floaters at week 1 was reduced, as Dora misunderstood 2 patients. In addition, the κ agreement for the symptom “pain” at week 1 was low due to the low prevalence of pain symptoms in this cohort (n=1). When comparing Dora-NL1 to regular care at week 1, no clinical issues were missed by Dora-NL1, resulting in 100% sensitivity, while the specificity was 41.86% due to the high rate of false positives.

### Comparison With Prior Work

Previous studies have demonstrated that telephone follow-ups are a safe method for identifying significant issues after cataract surgery [9,18,19]. In this study, 3 patients had inflammatory cells, which required a management change during their 4-week hospital visit. These complications were detected during slit lamp examination, highlighting a limitation of telephone consultations, which may fail to identify such issues in asymptomatic patients. Postoperative inflammatory cells usually peak at day 1 after surgery and may take up to 3 months to resolve [20]. Studies have demonstrated a correlation between prolonged intraocular inflammation and an increased likelihood of developing cystoid macular edema (CME), suggesting a threshold beyond which extended corticosteroid treatment is advisable [21]. However, randomized controlled trials comparing corticosteroid treatments versus placebo for inflammation after cataract surgery have shown that only 2.7% to 3.8% of patients in the placebo groups developed CME within 3 months after surgery versus 1.4% to 3.8% in the corticosteroid-treated groups [22,23]. These findings pose the question of clinical relevance of the presence of inflammatory cells in asymptomatic patients who have no symptoms of red eye or ocular pain or decreased visual acuity due to clinically significant CME. Additionally, another patient in our cohort who required a management change at week 4 erroneously reported experiencing binocular diplopia while there appeared to be monocular diplopia in the contralateral eye. Notably, this symptom was also missed by the clinician-led call at week 1. The cases missed by Dora-NL1 at 4 weeks postoperatively highlight the limitations of this tool in detecting subclinical or asymptomatic complications. While some complications, such as mild inflammation, may resolve without intervention, others, such as retained lens material, could lead to more serious consequences, including chronic inflammation and secondary glaucoma, or corneal decompensation. It should, however, be noted that the Dora call provides safety net advice for the patient to seek review with new or worsening symptoms. Hence, this tool should be viewed as a complement to, rather than a replacement for, in-person follow-up visits.

The English Dora-R1 model has been investigated in the United Kingdom, showing it to be a safe alternative for cataract surgery follow-up, with automated calls conducted 3‐4 weeks postoperatively and reviewed by an ophthalmologist [11,15]. The sensitivity of care management decision-making was 93.75% (95% CI 84.76-98.27), and the specificity was 86.26% (95% CI 79.16-91.56), with an accuracy of 88.72% (95% CI 93.42-92.79) [11]. These results are consistent with our findings in Tables2  4. In the UK cohort, 10 patients who had been discharged following a Dora-R1 call—based on both Dora’s and the supervising clinician’s decision—experienced unexpected management changes at their 4-week routine hospital visit. These patients were either asymptomatic or exhibited mild symptoms that were not reported during the Dora-R1 call. Among them, 3 were diagnosed with CME, 5 presented with inflammatory cells, 1 experienced a refractive surprise, and 1 developed early posterior capsular opacification [11]. These findings are consistent with our results, as the majority of cases not detected via the telephone call were in asymptomatic patients with inflammatory cells in the anterior chamber.

The NPS for Dora-R1 ranged from +45 to +51, with mean scores of 8.55 (SD 1.67) and 8.59 (SD 2.05), respectively. The overall TUQ mean score was 3.96 (SD 0.47; 95% CI 3.76-4.16) [11,15]. The mean NPS at 3 weeks for the Dora-R1 was compared with that at 4 weeks postoperatively, yielding a statistically significant mean difference of −1.21 (SD 0.27; *P*<.0001). Compared to UK studies, both NPS and TUQ scores in our Dutch cohort were lower. Several factors may account for this difference. First, the study design differed: while the UK participants received either only a Dora-R1 call or a Dora-R1 call along with a hospital visit at week 4, all Dutch participants received automated Dora-NL1 calls in addition to standard postoperative care, including telephone consultations and hospital visits. Consequently, patient expectations may have been shaped by direct comparisons between Dora and conventional care, potentially leading to more critical evaluations. Additionally, the Dora-NL1 model was adapted from the pretrained English version but relied on more limited Dutch natural language processing resources at the time of development. This may have affected the performance of both the text-to-speech and speech-to-text functions, resulting in less fluent and less natural user interactions [24]. Furthermore, cultural attitudes toward artificial intelligence (AI) and digital communication in health care may have contributed. Dutch patients may place a higher value on personal interaction and may be more reserved in expressing satisfaction with automated systems, particularly when these are perceived as less empathetic or less competent communicators. Future cross-cultural studies using harmonized protocols are needed to clarify the extent to which these differences are attributable to cultural, technical, or methodological factors.

### Limitations

Limitations of this study mainly included the potential selection bias. There were eligible patients who decided not to participate due to the expected difficulties of the Dora-NL1 call or who strongly opposed eHealth solutions in general. This population may not represent the broader cataract population, limiting generalizability. In addition, this is a mono-center study, which reduces the applicability to other health care facilities in the Netherlands, as there is a high variability in practice patterns of the postoperative cataract care pathway. Additionally, as all participants received both the Dora-NL1 and standard follow-up (clinician phone calls and in-person visits), the study is subject to performance bias. The awareness of being monitored by multiple systems may have influenced patient behavior, which may have influenced the outcomes.

Moreover, symptom-based systems like Dora-NL1 cannot detect structural or subclinical complications, which may require in-person assessment. Therefore, this tool is suitable as a complementary screening tool, replacing regular telephonic consultations for complication screening, but not as a substitute for in-person postoperative assessments.

### Future Implementation

Our findings indicated that at the 1-week follow-up, the Dora-NL1 model accurately identified 41.4% (n=36) of patients as having “no clinical concerns.” This suggests that approximately 4 of 10 patients could avoid clinician callbacks, potentially enhancing health care efficiency and reducing costs. Telephone reviews have been shown to be a safe time- and cost-saving alternative to short-term hospital visits after routine surgeries, with high patient satisfaction [18,19,25]. Moreover, automated calls allow clinicians to save time and focus on other care tasks while ensuring a standardized approach to symptom evaluation and decision-making. This consistency reduces variability, improves the quality of patient care, and facilitates the collection and comparison of patient outcomes. In the UK cohort, the average staff cost savings amount to US $47.78 per patient compared to regular care [11]. Further research is necessary to evaluate the potential cost-effectiveness in the Netherlands.

To implement automated calls in routine postoperative cataract care, several practical issues must be considered, particularly the significant variability in postoperative care across clinics and countries [26]. In some clinics, patients may not receive follow-up visits, as research indicates that for those undergoing uncomplicated cataract surgery without pre-existing ocular comorbidities, follow-up can be safely deferred to 2 to 4 weeks after surgery without significant differences in visual outcomes [27,28]. However, automated phone calls for interim check-ups could enhance patients’ confidence and reassurance during their recovery without straining health care resources. Despite this potential benefit, the current Dutch cataract guidelines do not yet recommend remote care for the final 4-week postoperative follow-up due to the limited evidence regarding its safety and the necessity of visual acuity and refraction assessments for the mandatory quality registry of cataract surgeries in the Netherlands [29]. Further development of the Dora-NL1 model is expected to improve its accuracy and expand its applicability.

### Conclusions

Based on the results of this study, we can conclude that the Dutch AI-based cataract follow-up call system, Dora-NL1, is a safe alternative for telephone consultations in the postoperative cataract care pathway and is suitable as a screening tool for postoperative complications during the follow-up of cataract surgery. However, as this tool only captures subjective information, it is not able to fully replace final 4-week in-person hospital visits after cataract surgery in the Netherlands. Future research is necessary to investigate the practical implications and cost-effectiveness of this AI-driven teleconsultation system.

## Supplementary material

10.2196/72574 Multimedia Appendix 1 Dora decision algorithm and symptom classification.

10.2196/72574 Multimedia Appendix 2 Agreement between Dutch and English models across different patient scenarios.

10.2196/72574 Multimedia Appendix 3 Outcomes of Telehealth Usability Questionnaire.

## Abbreviations

AI: artificial intelligence
CME: cystoid macular edema
IOL: intraocular lens
NPS: Net Promoter Score
TUQ: Telehealth Usability Questionnaire
