# Supplementary material for: Optimizing the Postcataract Patient Journey Using AI-Driven Teleconsultation: Prospective Case Study
Source: JMIR Form Res. 2025 Aug 18;9:e72574. doi: 10.2196/72574 (PMC12360671; doi:10.2196/72574)
Supplement: Multimedia Appendix 1 [file formative-v9-e72574-s001.pdf]

Decision-making Dora call

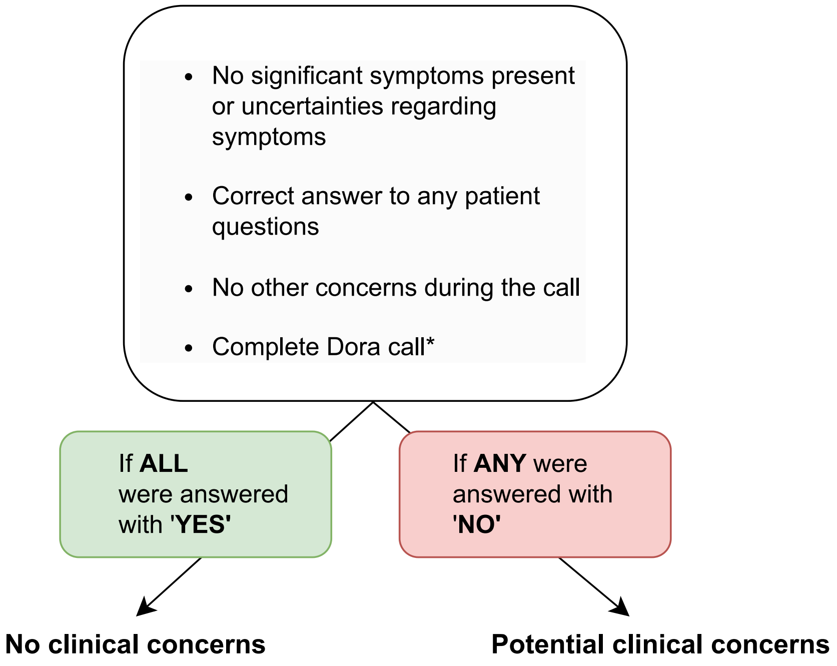

*A complete call was defined as completing all clinically relevant sections, including symptom assessments and patient questions.

Symptom classification

Symptom	Not significant	Signficant
Red eye	• No redness<br>• Only red in the corner by the nose<br>• Occasional redness in the eye<br>• Eye was red, but not anymore	• Eye is red all over<br>• Progressively worsening redness
Pain	• No pain<br>• Pain described as gritty or dry eye complaints<br>• Pain has resolved<br>• Pain only when using eye drops	• Painful all the time<br>• Worsening pain<br>• Deep or severe pain
Vision issues	• Patient is satisfied with vision<br>• Good vision<br>• Blurred reading vision, but good distance vision	• Patient has concerns about vision<br>• Worsening vision<br>• Unable to see clearly in the distance<br>• Distorted vision
Flashing lights	• No flashing lights in vision	• Any flashing lights in vision
Floaters	• No floaters<br>• Pre-existing floaters	• Any new floaters
