# Supplementary material for: Optimizing the Postcataract Patient Journey Using AI-Driven Teleconsultation: Prospective Case Study
Source: JMIR Form Res. 2025 Aug 18;9:e72574. doi: 10.2196/72574 (PMC12360671; doi:10.2196/72574)
Supplement: Multimedia Appendix 2 [file formative-v9-e72574-s002.docx]

| **Scenario/Component** | **Redness** | **Pain** | **Vision issue** | **New Floaters** | **Flashing Lights** | **Decision** |
| --- | --- | --- | --- | --- | --- | --- |
| **1. Asymptomatic**  *Answers*  *Dutch model*  *English model* | No | No | No | No | No | No review |
|  | No | No | No | No | No | No review |
|  | No | No | *Missing* | No | No | Review |
| **2. Dry eye**  *Answers*  *Dutch model*  *English model* | Yes | No | No | No | No | Review |
|  | Yes | No | No | No | No | Review |
|  | Yes | No | No | No | No | Review |
| **3. Uveitis**  *Answers*  *Dutch model*  *English model* | Yes | Yes | Yes | No | No | Review |
|  | Yes | Yes | Yes | No | No | Review |
|  | Yes | Yes | Yes | No | No | Review |
| **4. Vitreoretinal problem**  *Answers*  *Dutch model*  *English model* | No | No | No | Yes | Yes | Review |
|  | No | No | No | Yes | Yes | Review |
|  | No | No | No | Yes | Yes | Review |
| **5. Endophthalmitis 1**  *Answers*  *Dutch model*  *English model* | Yes | Yes | Yes | Yes | No | Review |
|  | Yes | Yes | Yes | Yes | No | Review |
|  | Yes | Yes | Yes | Yes | No | Review |
| **6. Endophthalmitis 2**  *Answers*  *Dutch model*  *English model* | Yes | Yes | Yes | No | No | Review |
|  | Yes | Yes | Yes | No | No | Review |
|  | Yes | Yes | Yes | No | No | Review |
| **7. Macular edema**  *Answers*  *Dutch model*  *English model* | No | No | Yes | No | No | Review |
|  | No | No | Yes | No | No | Review |
|  | No | No | Yes | No | No | Review |
| **8. Refractive surprise**  *Answers*  *Dutch model*  *English model* | No | No | Yes | No | No | Review |
|  | No | No | Yes | No | No | Review |
|  | No | No | Yes | No | No | Review |
| **9. High IOP**  *Answers*  *Dutch model*  *English model* | No | Yes | No | No | No | Review |
|  | No | Yes | No | No | No | Review |
|  | No | Yes | No | No | No | Review |
| **10. Subconjunctival hemorrhage in corner of the eye**  *Answers*  *Dutch model*  *English model* | No | No | No | No | No | No review |
|  | No | No | No | No | No | No review |
|  | No | No | No | No | No | No review |
| **11. Corneal edema**  *Answers*  *Dutch model*  *English model* | No | No | Yes | No | No | Review |
|  | No | No | Yes | No | No | Review |
|  | No | No | Yes | No | No | Review |
| **12. Old floaters**  *Answers*  *Dutch model*  *English model* | No | No | No | No | No | No review |
|  | No | No | *Missing* | No | No | No review |
|  | No | No | No | No | No | No review |
