# Supplementary material for: Optimizing the Postcataract Patient Journey Using AI-Driven Teleconsultation: Prospective Case Study
Source: JMIR Form Res. 2025 Aug 18;9:e72574. doi: 10.2196/72574 (PMC12360671; doi:10.2196/72574)
Supplement: Multimedia Appendix 3 [file formative-v9-e72574-s003.docx]

| **Component** | **Questionnaire item** | **Mean ± SD** |
| --- | --- | --- |
| **Usefulness** | Q1 ‘Telehealth improves my access to healthcare services’ | 3.11 ± 0.87 |
|  | Q2 ‘Telehealth saves me time traveling to a hospital or specialist clinic’ | 3.80 ± 1.11 |
|  | Q3 ‘Telehealth provides for my healthcare needs’ | 3.05 ± 0.97 |
| **Ease of use & Learnability** | Q4 ‘It was simple to use this system’ | 3.89 ± 0.96 |
|  | Q5 ‘It was easy to learn to use the system’ | 3.94 ± 0.88 |
|  | Q6 ‘I believe I could become productive quickly using this system’ | 2.98 ± 1.00 |
| **Interface quality** | Q7 ‘The way I interact with this system is pleasant’ | 2.99 ± 1.19 |
|  | Q8 ‘I like using the system’ | 3.00 ± 1.16 |
|  | Q9 ‘The system is simple and easy to understand’ | 3.74 ± 0.97 |
|  | Q10 ‘The system is able to do everything I would want it to be able to do’ | 2.72 ± 1.03 |
| **Interaction quality** | Q11 ‘I could easily communicate using the telehealth system’ | 3.10 ± 1.08 |
|  | Q12 ‘I could hear everything clearly using the telehealth system’ | 3.97 ± 0.91 |
|  | Q13 ‘I felt I was able to express myself effectively’ | 2.78 ± 1.14 |
|  | Q14 ‘Using the telehealth system, it can see the clinician as well as if we met in person' | 2.46 ± 1.07 |
| **Reliability** | Q15 ‘I think the visits provided over the telehealth system are the same as in-person visits’ | 2.79 ± 1.11 |
|  | Q16 ‘Whenever I made a mistake using the system, I could recover easily and quickly’ | 3.18 ± 0.83 |
| **Satisfaction & Future use** | Q17 ‘I feel comfortable communicating with the clinician using the telehealth system’ | 3.07 ± 1.09 |
|  | Q18 ‘Telehealth is an acceptable way to receive healthcare services’ | 3.06 ± 1.13 |
|  | Q19 ‘I would use the telehealth services again’ | 3.05 ± 1.25 |
|  | Q20 ‘Overall, I am satisfied with the telehealth system’ | 3.13 ± 1.20 |
